# Undiagnosed Acquired Hemophilia A: Presenting as Recurrent Gastrointestinal Bleeding

**DOI:** 10.7759/cureus.10188

**Published:** 2020-09-01

**Authors:** Arya Mariam Roy, Aisha Siddiqui, Anand Venkata

**Affiliations:** 1 Internal Medicine, University of Arkansas for Medical Sciences, Little Rock, USA; 2 Internal Medicine, American University of Antigua, New York, USA; 3 Pulmonary and Critical Care Medicine, University of Arkansas for Medical Sciences, Little Rock, USA

**Keywords:** acquired hemophilia a, gastro intestinal bleeding, bleeding disorders, gastroenterology and endoscopy, rituximab, feiba

## Abstract

Acute gastrointestinal bleeding (GIB) is a frequently encountered medical emergency and it can be life-threatening depending on the etiology and the clinical condition of the patient. The most common causes of GIB are peptic ulcer disease, aspirin-induced gastritis, variceal hemorrhage, esophagitis, neoplasms like gastric cancer. Acquired hemophilia causing acute gastrointestinal bleed is extremely rare and only a few cases are reported worldwide. Acquired hemophilia A (AHA) is a rare disorder caused by the production of autoantibodies that inactivates clotting factor VIII. We present a case of upper gastrointestinal bleed due to AHA which was undiagnosed for two years. A 74-year-old patient with a history of myasthenia gravis, presented with anemia, and GIB. She underwent multiple endoscopies without a clear bleeding source. Coagulation studies showed isolated activated partial thromboplastin time prolongation which was not corrected by mixing study. Factor VIII activity was low and Bethesda titer showed elevated inhibitor levels. Factor Eight Bypassing Agent, recombinant factor VIIa, and steroids were given to control bleeding. Her clinical condition worsened, and she passed away. Elderly patients presenting with an undiagnosed source of GIBs, inconclusive endoscopic studies should be evaluated for acquired coagulopathies, especially in those with a history of autoimmune diseases and malignancies. Prompt diagnosis and treatment are warranted as it carries a high mortality.

Part of the case presentation was presented as an abstract at a regional conference

## Introduction

Acute gastrointestinal bleeding (GIB) is a frequently encountered medical emergency, characterized by hematemesis, melena, or hemodynamic instability [1]. The most common etiologies of acute GIB are peptic ulcer disease, variceal hemorrhage, Mallory-Weiss tear, gastric malignancy, or non-steroidal anti-inflammatory drugs (NSAIDs) and aspirin-induced gastritis [2]. Cases of acute GIB secondary to acquired hemophilia A (AHA) are exceedingly rare and only a few cases have been reported worldwide. AHA is a rare bleeding disorder caused by autoantibodies against clotting factor VIII (FVIII) and predisposes to life-threatening hemorrhage [3]. The incidence of AHA is 1.2-1.48 cases per million/year [4] and occurs most commonly in the elderly population. AHA is seen associated with malignancy, autoimmune diseases, pregnancy, drugs, and blood transfusions. However, up to 50% of reported cases remain idiopathic [5]. Unlike joint bleeds that characterize congenital hemophilia A, AHA manifests as spontaneous hematomas and extensive bruising, epistaxis, rarely GIB, and intracranial hemorrhage may occur [3,5,6]. Reported mortality is between 6.2% and 44.3% in the year following the diagnosis. The high mortality warrants prompt clinical detection and treatment. It is also highly recommended to follow up these patients closely as there is a high chance for relapse [4]. Part of the case presentation was presented as an abstract at a regional conference.

## Case presentation

**Figure 1:**
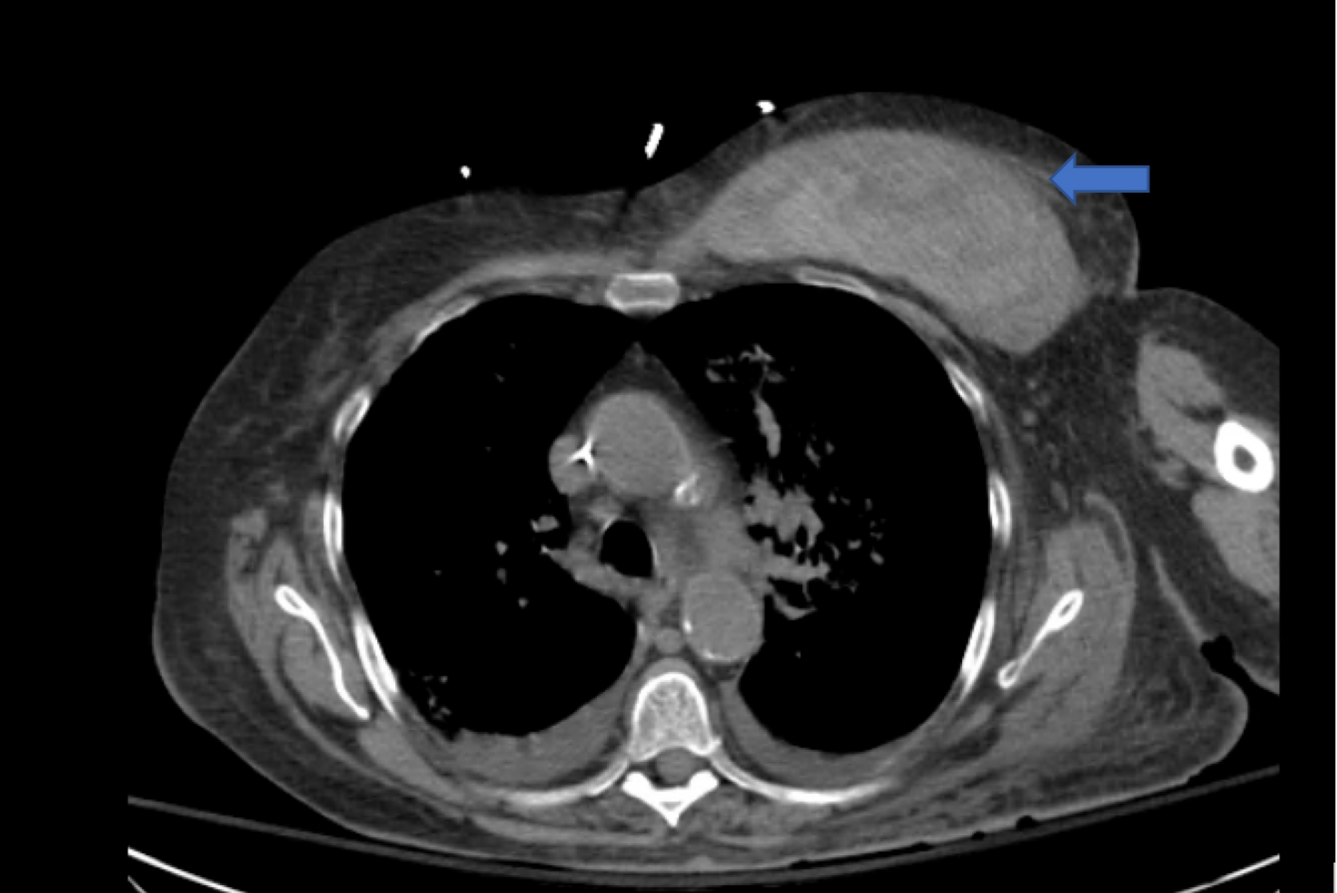
CT angiogram showing left pectoral hematoma

A 74-year-old Caucasian female with myasthenia gravis admitted with melena and a hemoglobin of 5.8 g/dl. Her history was significant for small bowel perforation requiring bowel resection and anastomosis two years before the current presentation followed by multiple admissions for anemia treated with blood transfusions and iron supplementations. She did not have any personal or family history of malignancies or bleeding disorders. She was diagnosed with myasthenia gravis a year before the current presentation and was started on pyridostigmine and high dose steroids initially. The steroids were tapered down to prednisone 5 mg daily, six months before the current presentation. She reported non-compliance with her medications for myasthenia gravis. One month before the current presentation, she had an episode of life-threatening acute gastrointestinal bleeding; however, endoscopy did not reveal a clear bleeding source, and the cause was attributed to erosive esophagitis. During the current admission for melena, both endoscopy and colonoscopy failed to show active bleeding. Tagged red blood cell scan, CT angiogram (CTA), capsule endoscopy showed oozing from the bowel anastomotic area. Surgical exploration was deferred given her multiple comorbidities. Later embolization of the bleeding vessel at the site of anastomosis was done by interventional radiology. One week after the procedure, she started to have melena and CTA demonstrated active extravasation again from the anastomotic area. Coagulation studies were performed in the setting of repeated bleeding and showed isolated activated partial thromboplastin time (APTT) prolongation which was not corrected by mixing study. Her FVIII activity was markedly reduced to <1% and factor IX activity was normal. Prothrombin time, fibrinogen, von Willebrand factor, lupus anticoagulant, malignancy workup, and hepatitis panel were normal. Bethesda titer showed elevated inhibitor levels at 91 Units. Steroids and rituximab were administered, and she was discharged with a plan of weekly rituximab for four weeks. She was readmitted two days after the discharge with spontaneous chest wall hematoma. CTA showed a large left pectoral hematoma measuring 14 x 13 x 5 cm with active extravasation (Figure 1). Factor Eight Inhibitor Bypassing Agent (FEIBA), recombinant factor VIIa, and steroids were given to control active bleeding. Clinically patient had stability of bleeding. However, during her subsequent hospital course, she acutely became unresponsive and resuscitative efforts proved to be unsuccessful, and the patient passed away. Intracranial hemorrhage was considered as a possible cause for her sudden death; however, an autopsy was not performed according to her family’s wishes.

## Discussion

Acute GIB can be life-threatening depending on the etiology and the clinical condition of the patient, and upper GIB is generally characterized by hematemesis or melena. Peptic ulcer disease, the most common cause of upper GIB (28%-59%), is associated with the use of NSAIDs and Helicobacter pylori infection. Variceal hemorrhage, aspirin-induced gastritis, steroid use, Mallory-Weiss tears, esophagitis, erosive gastritis/duodenitis, vascular ectasias, neoplasms like gastric cancer, and Dieulafoy’s lesions are other frequently encountered etiologies for upper GIB [1,2,7]. However, acquired bleeding disorders are an extremely rare cause of gastrointestinal bleeding. As such, we presented a case of upper GIB due to AHA which was undiagnosed for two years.

AHA is a rare disorder caused by the production of autoantibodies that inactivate clotting factors. In AHA, autoantibodies are directed against clotting factor VIII, and in acquired hemophilia B, autoantibodies inactivate or decrease levels of clotting factor IX [8]. In up to 50% of patients with AHA, an underlying medical condition can be identified, including autoimmune diseases (rheumatoid arthritis, multiple sclerosis, systemic lupus erythematosus inflammatory bowel disease, etc.), solid tumors, lymphoproliferative malignancies, and pregnancy [9]. However, in the rest of the patient population, a specific cause cannot be identified. The most encountered demographics of AHA are patients with a median age of 70 years and a small population of post-partum women who develop factor VIII inhibitors in association with pregnancy.

The clinical picture often includes spontaneous hemorrhage into skin, muscles, soft tissues, mucous membranes, unlike the joint bleeds that are seen in congenital hemophilia. It sometimes presents with gastrointestinal bleed, intracranial bleeds or prolonged bleeding after surgical procedures. AHA patients tend to experience more severe bleeding episodes than those with congenital hemophilia [10]. Cases of AHA are often undiagnosed or misdiagnosed because affected individuals do not have a personal or family history of bleeding. Further investigation is warranted when patients present with recurrent bleeding with no prior history of coagulopathy, and an unexplained and isolated prolonged aPTT; this is especially true for the at-risk demographic group.

Our patient was presented with anemia and gastrointestinal bleeding. She initially underwent multiple endoscopies without a localizing clear bleeding source and had chronic iron deficiency anemia with an etiology that was never diagnosed accurately. She remained undiagnosed for almost two years due to the rarity of acquired hemophilia presenting as a GIB. Her history of myasthenia gravis, an autoimmune disease is postulated to have precipitated the development of autoantibodies against FVIII, and also her history of noncompliance with steroids might have played a role in the exacerbation of acquired hemophilia. Elderly patients presenting with an undiagnosed source of GI bleeds, inconclusive endoscopic studies, and severe anemia should be evaluated for acquired coagulopathies, this is especially vital in those with the history of autoimmune disease and malignancies.

In a suspected case, if the aPTT is prolonged with normal prothrombin time, a mixing study should be performed. Failure to correct the aPTT in the mixing study indicates the presence of coagulation inhibitors which warrants checking of the clotting factor activity levels and factor inhibitor titer (Bethesda assay). A higher Bethesda titer indicates the stronger inhibition of anticoagulation [11,12]. Management of AHA involves primarily controlling the acute hemorrhage and eradication of the antibodies. In cases of bleeding with low inhibitor titers (<5 BU), FVIII replacement can be given [13]. For life-threatening bleeding with high inhibitor titer, factor bypassing agents such as FEIBA, recombinant activated human factor VIIa (rFVIIa) is the treatment of choice [14]. For the elimination of the antibodies glucocorticoids alone or in combination with rituximab or cyclophosphamide are primarily used. All patients should be closely followed up as relapses are common [15].

## Conclusions

AHA should be considered and coagulation studies should be done as a part of the initial workup when elderly patients present with recurrent bleeding and inconclusive endoscopic intervention especially in the setting of having a coexistent autoimmune disease. Prompt diagnosis and timely treatment are vital as this can lead to fatal hemorrhages. Early initiation of factor-bypassing agents and adherence to immunosuppressive agents can be lifesaving and can prevent relapses.

 Part of the case presentation was presented as an abstract at a regional conference.
